# Aggressive subtypes in basal cell carcinomas might need different treatment and follow-up due to the higher risk of surgically uncontrollable recurrences

**DOI:** 10.1007/s00417-025-06807-8

**Published:** 2025-03-26

**Authors:** Svenja Rebecca Sonntag, Rebecca Beach, Stefanie Gniesmer, Joyce Tohme, Salvatore Grisanti, Armin Mohi, Sara Hsin-Yi Yang, Vinodh Kakkassery

**Affiliations:** 1 Department of Ophthalmology, University of Lübeck, Medical Center Schleswig-Holstein, Campus Lübeck, Ratzeburger Allee 160, 23538 Lübeck, Germany; 2 Department of Dermatology, University of Lübeck, Medical Center Schleswig-Holstein, 23538 Lübeck, Germany; 3 Department of Ophthalmology, Clinic Chemnitz, 09116 Chemnitz, Germany

**Keywords:** Basal cell carcinoma, BCC, Recurrence, Eyelid tumor, Aggressive subtype

## Abstract

**Purpose:**

Basal cell carcinoma (BCC) is the most frequent malignant tumor of the eyelid and recurrences of BCC may lead to massive destruction of the orbital region. The objective of this study was to evaluate predictors for surgically difficult-to-control or uncontrollable recurrences.

**Methods:**

All BCCs of the periorbital region treated in the Department of Dermatology or Ophthalmology between 2011 and 2021 were included in a retrospective single center study and divided into a group of primary BCCs (pBCCs) and a group of recurrent BCCs (rBCCs). The following risk factors were compared between the two groups using the Chi^2^ test: tumor localization, histological subtype and presence of R1 situation. Furthermore, difference in severity of reconstruction between pBCCs and rBCCs was analyzed. *P*-value < 0.05 was considered statistically significant.

**Results:**

Data from 474 pBCCs and 33 rBCCs were included in this retrospective analysis. Both R1 status (*p* < 0.001) and aggressive subtype (*p* = 0.028) were significant risk factors for recurrence.

The two most frequent reasons for R1 were the patient’s rejection of further surgical intervention (*n* = 4) and the fact that the surgery was not performed at a specialized center (*n* = 6).

In 10 of the 33 rBCCs, a further recurrence occurred despite R0 status and all 10 cases showed an aggressive subtype (*p* = 0.020). In all BCCs with R1 status, there was no significant difference in the recurrence rate regarding the subtype.

**Conclusion:**

Our results show the impact of incomplete tumor resection and aggressive subtype on patient outcome after BCC surgery. We suggest that the aggressiveness of the BCC may be the precondition for multiple recurring BCCs. Furthermore, especially patients who underwent surgery outside our Departments showed R1 situations and rBCCs. Therefore, personalized treatment and follow-up care as well as efforts to avoid high-risk recurrences with aggressive subtypes are necessary to improve long-term success after surgery and should be conducted by a specialized center.

## Key messages

***What is known***:

• Basal cell carcinoma is the most frequent malignant eyelid tumor and can lead to massive destruction of the orbital region, especially if we have a recurrence.

• There are already well-known risk factors for recurrence as the histological subtype, R0/R1 situation or tumor size.

***What is new***:

• Our study compares those risk factors between patients with one recurrence and patients with multiple recurrences

• Whereas for the primary recurrence the R1 situation is the most significant risk factor, multiple recurrences seem to rely on an aggressive subtype.

• Besides this difference in risk factor significance, the surgical reconstruction was evaluated and showed significantly more complex surgical procedures for patients with recurrences.

## Introduction

Basal cell carcinoma (BCC) is the most common malignant tumor in humans in Central Europe and worldwide [1]. The incidence in Germany is around 200/100,000 persons per year, while it varies greatly worldwide with a maximum of 1,000/100,000 persons per year in Australia and a minimum of 1/100,000 in Africa [2]. In addition, the incidence is continuously increasing from year to year due to higher UV exposure [3]. It is assumed that BCC of the eyelid region accounts for up to 20% of all BCCs [4, 5].

Surgical resection with histological control for a total removal of the tumor is still the gold standard of treatment, as it has the lowest rates of recurrence [6]. However, the recurrence rate is highly dependent on several risk factors. These risk factors include histological subtype and free tumor margins (R0 situation) [7, 8]. The histological subtypes can be divided into more aggressive forms such as sclerodermiform, infiltrating, metatypical or micronodular BCC and less aggressive forms as superficial, nodular, cystic or fibroepithelial BCC [1].

Another risk factor for recurrence might be tumor localization, which is controversially discussed in literature [9–11], as well as tumor diameter. Tumors in the periorbital zone with a basal diameter larger than 6 mm have been classified as high-risk tumors for recurrence [13].

A major problem of BCC recurrences is that they may often lead to even larger eyelid defects, infiltration of the lacrimal ducts or of the muscles and the neighboring orbital bones [12, 13]. In some cases, BCCs recur more than once and surgical tumor control can no longer be achieved. Adjuvant therapy with radiation or Hedgehog signalling pathway inhibitors might be more successful, but there are still patients with difficult-to-control or uncontrollable tumors [14, 15].

Although there are many studies analyzing the risk factors of recurring BCCs [6, 7, 11], no differentiation has been made between rBCCs with only one recurrence and rBCCs with multiple recurrences and subsequent alternative therapy via radiation or systemic treatment.

The objective of the study was therefore to analyze not only the risk factors for BCC recurrence, but also the risk factors for surgically difficult-to-control or uncontrollable recurrences.

## Methods

### Study design

In this monocentric, retrospective clinical study the histological database in the Dermatopathology Lab, Department of Dermatology of the University Medical Center Schleswig–Holstein, Campus Lübeck, was searched for all BCCs of the periorbital region treated in the Department of Ophthalmology or the Department of Dermatology between 2011 and 2021. The study was positively reviewed by the ethics committee of the University of Lübeck (No. 2022–421) and conducted in accordance with the ethical standards stated in the Declaration of Helsinki.

**Fig. 1:**
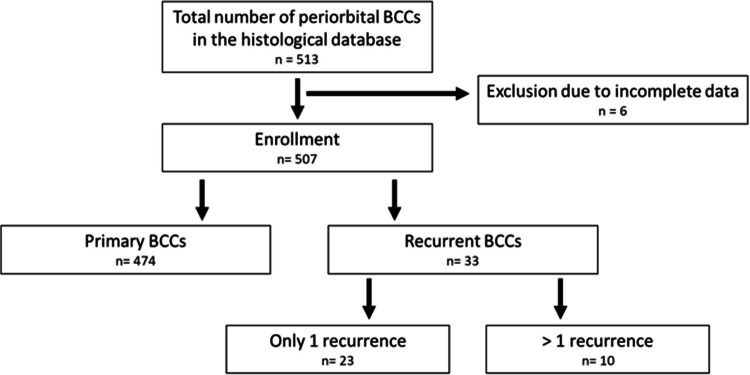
Flowchart of the patient selection

A total number of 513 cases were documented in the histological database, of which 474 primary BCC (pBCC) and 33 recurrent BCC (rBCC) were enrolled in the study (Fig. 1). In the 507 cases included in our study, data was available in nearly all categories. Only 6 patients were excluded from the study due to missing data.

Follow-up was conducted regularly at our clinic 3 months after the surgical procedure. Further follow-ups were performed by an ophthalmologic practice and the patients were only sent to us if there were notable changes or a suspected recurrence.

### Data collection parameters

Both groups were characterized in terms of age, gender, tumor diameter and tumor thickness. Furthermore, the histological subtype, tumor location and R0 situation were recorded from the patient files in order to determine which parameters could promote recurrence. The histological subtype was further subdivided into infiltrating, metatypical, sclerodermiform and micronodular BCC. These four subtypes were categorized as aggressive forms of BCC as mentioned in the introduction. The other subtypes such as superficial, nodular, cystic and fibroepithelial BCC were classified as less aggressive forms. If more than one subtype was present (so-called mixed type), the aggressiveness of the BCC was only evaluated if all subtypes were uniform in this respect.

**Fig. 2:**
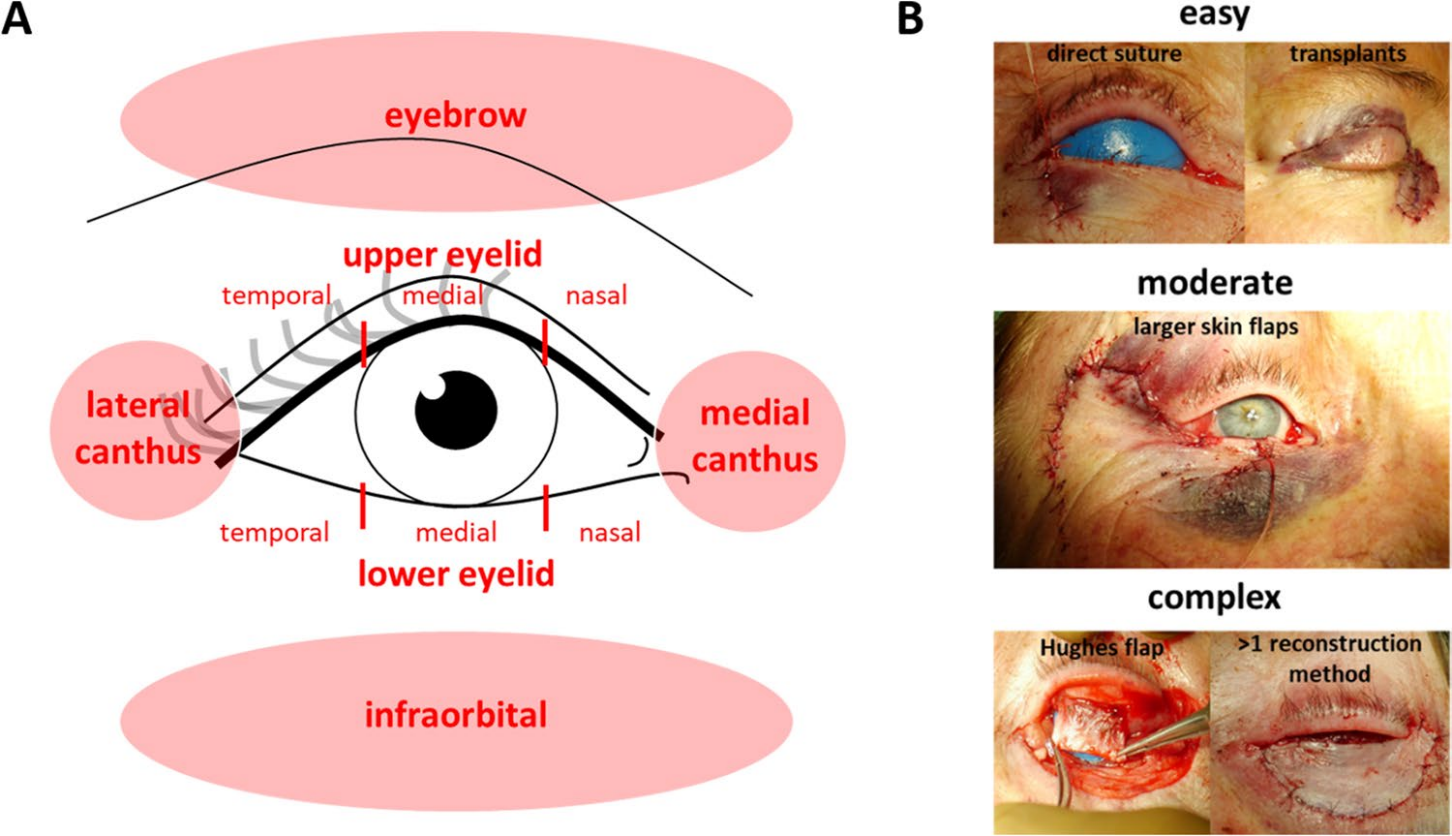
**A** Schematic depiction of the different eyelid localizations, which were used for statistical analysis. The 11 sub-localizations were further summarized in four groups consisting of the complete upper eyelid including the eyebrow region, the complete lower eyelid including the infraorbital region and the medial and lateral canthus (**B**) Exemplary images of eyelid reconstructions categorized for their severity. Direct sutures and free skin transplants were classified as easy (upper images), moderate reconstructions (image in the middle) used larger skin flaps and complex reconstructions (bottom images) included tarsus reconstruction or combination of more than one reconstruction technique

To determine localization, the third of the lower or upper eyelid, respectively as well as the medial or lateral canthus and the infraorbital or eyebrow region were indicated depending on the location (Fig. 2A).

Unfortunately, due to the retrospective design and the partial lack of information about the initial tumor, it was not always possible to determine whether the primary tumor was resected R0. Nevertheless, in addition to the aggressiveness of the BCC, the R0 situation was also analyzed and the necessary reconstruction method of the rBCCs was compared with that of the pBCCs in order to evaluate whether a recurrence is potentially associated with greater destruction and more complicated reconstruction.

Surgical reconstruction was categorized according to its severity (Fig. 2B). Direct closure with a simple eyelid or skin suture and skin grafts were defined as easy, all reconstructions with larger skin flaps, such as Tenzel flap, were defined as moderate, and all surgical procedures with more than one technique or greater need of tarsus reconstruction, such as a Hughes flap, were categorized as complex.

Finally, the group of rBCC was further subdivided into a group with only one recurrence and a group with more than one recurrence. Both groups were also further analyzed with regard to their histological subtype, R0/R1 situation and localization. The results of this analysis are presented at the end of the results section.

### Statistical analysis

The data above was analyzed by the statistical program Jamovi (version 2.4.14).

In addition to the descriptive evaluation, tumor thickness, histological subtype, gender, tumor localization and complexity of reconstruction were analyzed using the chi-squared test (X^2^-test). Age differences were evaluated using the Welsh’s test due to variance heterogeneity. Normality was proven via Shapiro–Wilk test. Comparisons were made between pBCCs and rBCCs as well as between rBCCs with one recurrence and rBCCs with more than one recurrence. A *p*-value of < 0.05 was set as the significant threshold.

The statistical analysis and the applied tests were reviewed in collaboration with the Institute of Medical Biometry and Statistics (IMBS) at the University of Lübeck.

## Results

### General data

As mentioned above, 507 cases were sufficiently documented in the histological database, including 33 cases with rBCCs (Table 1). 332 of the 507 patients underwent surgery in the Department of Ophthalmology and 175 in the Department of Dermatology. In all cases treated in the Department of Dermatology, the tarsus of the eyelids was omitted, and therefore no reconstruction of the eyelid margin was necessary.

**Table 1:** General data and tumor parameters

	pBCC	rBCC
Total number (n)	474	33
Age (mean ± SD)	75.1 ± 11.2	73.1 ± 10.1
Male (n)	206	12
Female (n)	268	21
Co- or preexisting skin cancer (n)	139	9
Tumor diameter (mean ± SD)	7.93 ± 5.26 mm	8.95 ± 7.03 mm
Tumor thickness (mean ± SD)	2.23 ± 1.08 mm	2.90 ± 2.59 mm

The mean age of the patients was 75.1 ± 11.2 years for the pBCC group and 73.1 ± 10.1 for the rBCC group. This result was not statistically significant (*p* = 0.298). The gender distribution was also not statistically significant with 206 male and 268 female pBCC cases and 12 male and 21 female rBCC cases (*p* = 0,276).

A statistical analysis regarding other co- or pre-existing malignant skin cancers showed no significant difference between the two groups (*p* = 0,802).

### Tumor parameters

Furthermore, all tumors were analyzed for tumor thickness and diameter (Table 1). The parameters measured for the rBCC group were not the same as the primary tumor resulting in the rBCC, because many of the tumors were surgically removed ex domo and the previous results could not be obtained. Therefore, the results cannot be seen as risk factors but as characteristics of both groups. There was no significant difference between the tumor thickness (*p* = 0.152) and the tumor diameter (*p* = 0.519). The mean tumor thickness in the pBCC group was 2.23 ± 1.08 mm and 2.90 ± 2.59 mm in the rBCC group, while the mean diameter was 8.95 ± 7.03 mm and 7.93 ± 5.26 mm, respectively. Thickness was measured by the pathologist, whereas tumor diameter was defined during the examination in the clinic.

### Tumor localization

Analyzing the tumor localization, there was no significant difference comparing all 11 sub-localizations (*p* = 0,276). Therefore, we also evaluated the distribution of tumors divided into lower eyelid (including infraorbital ones), upper eyelid (including eyebrow), medial and lateral canthus (Table 2). There was a significant difference of *p* = 0.035 between the pBCCs and rBCCs. In the pBCC group, 67.72% of the BCCs were located at the lower eyelid, 12.24% at the upper eyelid, 17.30% at the medial canthus, and 2.75% at the lateral canthus. In the rBCC group, we found only 54.55% located at the lower eyelid and 6.01% at the upper eyelid, but 30.30% at the medial canthus and 9.10% at the lateral canthus. These results suggest that the rBCCs are more common at the medial and lateral canthus. As we wanted to determine whether the significant difference was definitely due to the localization or whether there might be confounding parameters, we also compared the tumor diameter and thickness as well as histological subtype of all BCC between the different localizations. There was no significant difference in R1 situation or histological subtype, but the tumor diameter was significantly larger at both locations than in lower eyelid tumors (*p* < 0.001).

**Table 2:** Tumor localizations

Localization	pBCC	rBCC
Lower eyelid + infraorbital (n)	321	18
Upper Eyelid + eyebrow (n)	58	2
Medial canthus (n)	82	10
Lateral canthus (n)	13	3

### Histological subtype

Our analysis showed no significant difference between the individual subtypes in the two study groups (*p* = 0.335). For this reason, we analyzed the aggressiveness of the subtype according to the aggressiveness classification of the German BCC guideline [1]. However, there was still no significant difference between the two groups (*p* = 0.304), which was quite surprising regarding the known literature. In a third step, we therefore evaluated whether there is a significant statistical difference when excluding those rBCCs which were not completely resected in the first operation (R1 situation). The remaining rBCCs showed significant differences in terms of the aggressiveness of the tumor (*p* = 0.028). 49.57% of the pBCCs versus 75% of the rBCCs showed an aggressive tumor subtype (Table 3).

**Table 3:** Descriptive statistic of pBCCs and rBCCs regarding the histological subtype (upper part), the aggressiveness (middle) and the aggressiveness after exclusion of rBCC with R1 situation (lower part)

Histological subtype	pBCC	rBCC
Nodular (*n*)	142	8
Cystic (*n*)	6	0
Superficial (*n*)	10	3
Fibroepithelial (*n*)	1	0
Mixed (*n*)	29	2
Infiltrative (*n*)	120	14
Metatypical (*n*)	44	5
Micronodular (*n*)	4	0
Sclerodermifom (*n*)	13	0
Aggressiveness	pBCC	rBCC
Less aggressive (*n*)	171	11
Mixed (*n*)	22	1
Aggressive (*n*)	182	20
Aggressiveness (excluding rBCCs with R1)	pBCC	rBCC
Less aggressive (*n*)	171	5
Mixed (*n*)	22	1
Aggressive (*n*)	182	19

### Severity of the reconstruction

To evaluate whether reconstruction of the eyelids and periorbital region is more complicated after rBCC surgery, we categorized the surgical reconstruction method according to its severity (explanation in the introduction) and found a significant difference in reconstruction severity between pBCCs and rBCCs (*p* = 0.039). Unfortunately, some of the pBCC data was missing. Nevertheless, only 29.68% of the pBCCs required a complex reconstruction, while 46.66% of the rBCCs underwent a complex surgical intervention (Table 4).

**Table 4:** Severity of reconstruction

Severity of reconstruction	pBCC	rBCC
Complex (*n*)	138	14
Moderate (*n*)	159	4
Easy (*n*)	168	12

### Rate of recurrence depending on the R0/R1 situation

Only 33 of the 507 BCCs showed more than one BCC in the patient’s medical history, corresponding to a recurrence rate of 6.96%. Regarding the recurrence rate of the rBCCs, 10 of 33 rBCC recurred for a second time, which constitutes a recurrence rate of 30.30%.

For further analysis of the impact of R0/R1-situation on the recurrence rate, we examined the recurrence rate after R0 and R1 situation of pBCC and rBCCs, as well as of rBCCs with more than one tumor recurrence.

In total, 15 of all 507 BCCs had an R1 situation and 7 of these patients showed a tumor recurrence, corresponding to 46,67%. Thus, pBCC after R1 situation showed the highest rates of recurrence.

Dividing the R1 patients into groups with an aggressive or non-aggressive form, respectively, there was no significant difference between the recurrence rates (*p* = 0.741). In the group of rBCCs with more than one tumor recurrence, each of the 10 tumor patients had an aggressive histological subtype. More precisely, 9 of them were infiltrating BCCs and one metatypical. Two of those patients had Gorlin-Goltz-Syndrome and none of the 10 patients showed an R1 situation.

The two most frequent reasons for R1 situation were the patient’s rejection of further surgical intervention (*n* = 4) and that the surgery was not performed at a specialized center (*n* = 6).

## Discussion

About 90% of malignant tumors of the eyelid are BCCs [16]. Even though most of the tumors can be successfully treated with surgery, tumor recurrence remains a considerable problem.

It is already well-known, in addition to the statistically relevant parameters found in our study, that the recurrence rate also depends on the tumor size [17] and the different treatment options [18]. Tumors with a diameter of less than 2 cm generally show recurrence rates of about 10%, whereas tumors with larger diameters may recur in more than 40% of cases. With the different surgical approaches such as Moh’s micrographic surgery, resection with or without traditional extemporaneous pathology, the recurrence rates for tumors less than 2 cm range from 1.5% to 6%, 2% to 7% and 10% to 18% [17].

In our study, we determined a recurrence rate of 6.51% and a mean tumor diameter for both groups of about 8 mm. Total resection of the tumor was controlled by a modification of Moh’s micrographic surgery with histopathological margin assessment and further resection in R1 situations. It has been shown that this surgical technique does not yield significantly worse results compared to Moh’s micrographic surgery [18].

Interestingly, the German BCC Guideline mentions an even higher risk of recurrence in the periorbital region for tumors larger than 6 mm, which means that our study group seems to be at an even higher risk of recurrence than one might assume if we only consider a higher risk for tumors larger than 2 cm [1, 18].

Comparing our recurrence rates with a recent study by Dethmers et al. [7] with a similar study design, their study revealed a higher rate of recurrence for the pBCCs of 16.5% and for the rBCCs of 46.2%. The documented follow-up time was longer than in our study, but unfortunately no tumor size is documented for a better comparison. However, the difference in recurrence rates might be due to the higher rate of R1 resections (30 out of 240 patients) compared to our rate (15 out of 507 patients).

These results correspond to the highly significant risk factor of the R1 situation (*p* < 0.001) in our study. Therefore, the low number of cases with R1 situation might explain our lower recurrence rate compared to some other studies [7, 10, 19, 20].

Regarding the risk factors for recurrence which we determined in our study, the already established risk factor of R1 situation has been described in many studies and has been confirmed by ours [9, 21–23]. However, we also noticed that especially the patients treated outside our clinic showed R1 situation. In most cases, this was due to the limited surgical options ex domo after R1 situation.

The tumor localization as a risk factor is controversially discussed in the literature [7, 9, 11, 24]. We were able to demonstrate a significant difference with higher rates of rBCCs at the medial and lateral canthus compared to the group of pBCCs. In some studies it is discussed whether this significant difference concerning the medial canthus might be due to the R1 situation and the smaller safety margin of resections in order to preserve the lacrimal ducts [7, 9, 11, 24]. Examining the data of all BCCs in our study, there were no significant differences in terms of R1 situation or histological subtype between the different localizations, but the tumor diameter was significantly larger in both locations compared to the tumors at the lower eyelid (*p* < 0.001). Therefore, we assume that the significantly higher amount of rBCCs might rather be influenced by the tumor diameter and that the higher risk for rBCCs at the medial canthus described in literature might rather be due to confounding parameters such as R1 situation or tumor diameter.

The aggressive subtype as a further risk factor has also been described previously [11]. We were only able to show a significantly higher risk in aggressive subtypes after excluding BCCs with R1 situation. However, the rBCCs with more than one recurrence showed that the aggressive subtype is a clear risk factor for multiple recurrences, as there was a significant difference in the subtype depending on the number of recurrences within the rBCC group. All patients with more than one recurrence had an aggressive subtype, predominately the infiltrating subtype, and none of them had an R1 resection. Therefore, we assume that the most important risk factor of recurrent recurrences is the infiltrating subtype of the BCC.

In addition to the aggressiveness of the tumor itself, it has also been shown in a previous study that histological control is more difficult in infiltrating subtypes [10] and that there could be a higher number of patients incorrectly classified as R0 [25]. Furthermore, there could be other risk factors which may only be analyzed by immunohistochemical stainings or methods like next-generation sequencing.

An additional analysis in which we statistically compared the number of rBCC and divided the group of R1 resected tumors into groups with aggressive and non-aggressive subtypes, respectively, revealed no significant difference. Therefore, the surgeon may not be able to decide due to the subtype whether there is a higher risk of recurrence after R1 situation. However, if further resection destroys important surrounding tissue, the surgeon may decide, in collaboration with the patient, on an individual basis whether further resection should be performed. Indicators which may lead to the decision for watch-and-wait are the age of the patient, as many rBCC occur more than 5 years after the resection, a non-aggressive subtype, as the possible rBCC can be well controlled in the future, or the pathologist’s estimation of the quantity of the tumor rest (single cells vs. large tumor rest), as Auw-Haedrich et al. showed that only about 30% of re-excised tumors showed tumor cells in the tissue sections, which correlated with the rate of recurrence [8]. However, such parameters may only be an attempt to evaluate the individual patient’s risk of experiencing a rBCC, the best way to avoid rBCC will always be the total resection with free tumor margins. To reach this goal, it is necessary to include the patient in every decision and to explain the importance of potentially more than one excision.

Especially in R1 situations and in patients with multiple recurrences, strict follow-up might be the way to avoid difficult-to-control or uncontrollable recurrences. A larger study from Sartore et al. [26] showed that the probability of recurrence is elevated up to 2.7-fold depending on different risk factors. It is recommended that patients with higher risk of recurrence be followed every 3 months in the first year and then every 6 months for at least two further years. Patients without further risk for recurrence can be controlled annually.

Another study from Loone et al. [27] on BCC follow-up, not specifically for facial BCC, also states the burden of regular follow-up in outpatient clinics as well as the reduced patient compliance with routine follow-up. Therefore, it is highly important to explain the reason for the follow-up to the patient and we recommend that patients with higher risk for rBCC are followed in a specialist center.

One limitation of our study remains the small group of rBCCs for comparison, even though we have a larger data set than many other studies on periorbital BCCs [7, 21–23]. Additionally, our study results are limited by the lack of data for some of the tumors due to the retrospective study design. The tumor diameter was only documented for 346 cases. Another aspect which might lead to the different rates of recurrences compared to the literature could be the follow-up time, as short-term recurrence (< 5 years after surgery) only occurs in about half to two- thirds of recurring cases and all other recurrences occur later (> 5 years follow-up) [20, 22]. If the postoperative situation was good and we did not have a particularly high risk for recurrence, our patients were only controlled once in our clinic three months after reconstructive surgery. Further controls were performed ex domo. We assume that most of the patients would have been sent to our clinic again in case of recurrence, as it was done with the 33 recurrent cases. However, our recurrence rate might be underestimated due to unknown rBCC cases.

In summary, we were able to confirm that the R0 situation is highly recommended for all BCC and that the decision between further surgery or watch-and-wait in patients with R1 situation should be an individual, well-considered decision together with the patient, as we were not able to find definite hints to predict recurrence in such situation. If we decide to tolerate R1 situations, further controls after the R1 situation are highly important. The reason that BCCs show recurrent recurrences seems to be the aggressive subtype of the BCC or further parameters we have yet to establish. Strict follow-up is highly important here. Additional research should be done to evaluate further risk factors and possible therapeutic strategies, for example using immunohistochemical staining or next-generation sequencing.
